# Recent Advances in Biosensors Using Enzyme-Stabilized Gold Nanoclusters

**DOI:** 10.3390/bios15010002

**Published:** 2024-12-24

**Authors:** Myeong-Jun Lee, Jeong-Hyeop Shin, Seung-Hun Jung, Byung-Keun Oh

**Affiliations:** Department of Chemical and Biomolecular Engineering, Sogang University, Seoul 04107, Republic of Korea; audwns5413@sogang.ac.kr (M.-J.L.); ra7ine@sogang.ac.kr (J.-H.S.); shchng1998@sogang.ac.kr (S.-H.J.)

**Keywords:** gold nanocluster, enzyme, biosensor, optical, electrochemical

## Abstract

Recently, gold nanoclusters (AuNCs) have been widely used in biological applications due to their ultrasmall size, ranging within a few nanometers; large specific surface area; easy functionalization; unique fluorescence properties; and excellent conductivity. However, because they are unstable in solution, AuNCs require stabilization by using ligands such as dendrimers, peptides, DNA, and proteins. As a result, the properties of AuNCs and their formation are determined by the ligand, so the selection of the ligand is important. Of the many ligands implemented, enzyme-stabilized gold nanoclusters (enzyme–AuNCs) have attracted increasing attention for biosensor applications because of the excellent optical/electrochemical properties of AuNCs and the highly target-specific reactions of enzymes. In this review, we explore how enzyme–AuNCs are prepared, their properties, and the various types of enzyme–AuNC-based biosensors that use optical and electrochemical detection techniques. Finally, we discuss the current challenges and prospects of enzyme–AuNCs in biosensing applications. We expect this review to provide interdisciplinary knowledge about the application of enzyme–AuNC-based materials within the biomedical and environmental fields.

## 1. Introduction

In the realm of nanoscience and nanotechnology, the intricate properties of matter at the nanoscale have captivated scientists and innovators, leading to the discovery and exploration of novel materials with exceptional attributes [1,2,3]. Among these remarkable materials, gold nanoclusters (AuNCs) have emerged as a subject of intense investigation, offering a fascinating bridge between the macroscopic world and the quantum realm [4,5,6]. AuNCs, often referred to as “nano-gold”, exemplify the extraordinary changes that occur when materials are confined to dimensions in the order of nanometers. These clusters possess properties that stand apart from those of bulk gold and other nanomaterials due to their unique electronic structure and surface properties [7,8,9,10]. For example, AuNCs have molecule-like characteristics, such as discrete energy levels, size-dependent fluorescence, high conductivity, and strong photoluminescence, originating from their size approaching the Fermi wavelength of electrons between Au atoms and nanoparticles [11,12,13]. Along with these perfect properties, AuNCs have assets such as good biocompatibility and bioactivity and can be generated according to an eco-friendly production process [14,15,16]. For these reasons, AuNCs have been used and have demonstrated promising potential in a wide range of biological applications, such as biosensing, biolabeling, and bioimaging.

Although AuNCs have outstanding properties, there are still demerits and challenges involved in their use; for example, AuNCs must be stabilized to prevent self-agglomeration. Therefore, ligands such as dendrimers, peptides, nucleic acids, and proteins are required to stabilize AuNCs [17,18,19,20,21]. However, some of these ligands require complicated synthetic processing or pose harm to the environment (such as phenylethane thiol), and some AuNCs have shown low stability, fragility in terms of the pH, and poor water solubility, hindering their broader application.

Of many ligands, enzyme-stabilized gold nanoclusters (enzyme–AuNCs) in particular have gained interest due to their combination of the excellent chemical, electrical, and optical properties of AuNCs and the highly specific reactions of enzymes. Currently, enzymes are widely used for the specific detection of targets in many biosensing fields. However, challenges have also been encountered in optical and electrochemical detection, such as the need for additional materials for signal development and low conductivity, which reduces the sensitivity of electrochemical signals [22,23,24,25]. Efforts have been made to solve this problem by applying the excellent properties of enzyme–AuNCs to develop more convenient and sensitive biosensors.

**Figure 1:**
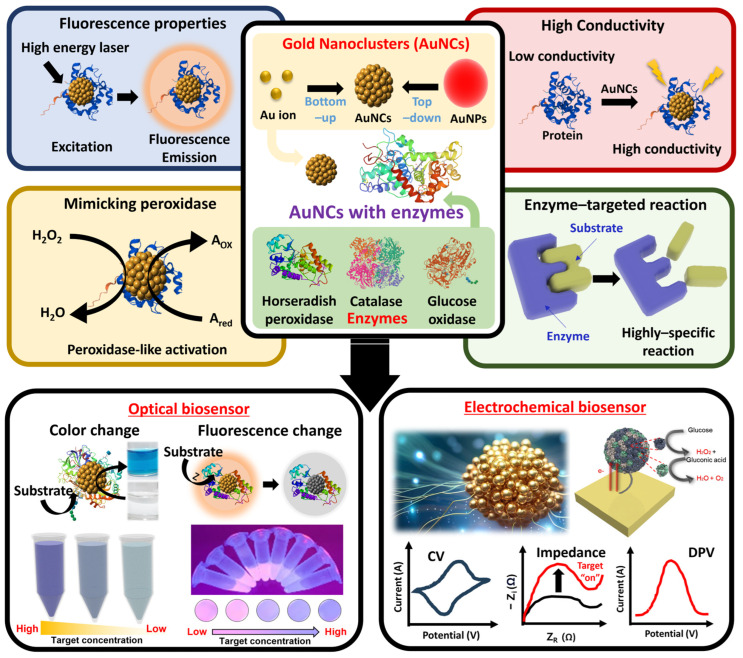
Schematic representation of different approaches of synthetic enzyme–AuNCs and their unique properties that are applicable on optical and electrochemical biosensors.

Although there are plentiful review papers on AuNCs, there are an insufficient number of review papers specializing in enzyme–AuNCs and their applications. Therefore, in this review, we focus on the properties of enzyme–AuNCs and recent examples of biosensors that apply these excellent properties in order to provide interdisciplinary knowledge about the use of enzyme–AuNC-based materials within the biomedical and environmental fields (Figure 1).

## 2. Preparation and Properties of Enzyme–AuNCs

### 2.1. Preparation of Enzyme–AuNCs

To produce ultrasmall AuNCs, several preparation protocols have been established, primarily falling into two main categories: “top–down” and “bottom–up” methods [26,27]. The top–down method for synthesizing nanoclusters involves breaking down larger metal structures into smaller nanoclusters. This approach contrasts with the bottom–up method, which builds nanoclusters atom by atom [28]. In the synthesis of AuNCs, “top–down” preparation protocols involve gradually etching gold nanoparticles (AuNPs) to produce AuNCs using etching agents such as HCl, dihydrolipoic acid, thiol groups, tertiary phosphines, mercaptoundecanoic acid, and glutathione (GSH) [29]. The top–down approach is the standard method for preparing nanomaterials, typically etching larger materials such as AuNPs in the presence of excess ligands to produce AuNCs. These core etching methods have been widely used to synthesize Au and AgNCs. Generally, this process takes place in an organic solvent, and the resulting nanoclusters tend to have better quantum yield and mono-dispersity than those synthesized via a “bottom–up” approach [30,31]. Although the top–down approach has been widely used to generate other ligand-capped AuNCs, further efforts are still required for protein-stabilized cases, as there are few reports in this area. This is likely because core etching is a harsh chemical process, and most proteins (including enzymes) lose their structure under these conditions, leading to a broad size distribution of the resultant particles [32]. These structural changes can greatly affect the performance of the enzyme–AuNCs because the enzyme’s functionality is significantly reduced by structural transformation. For this reason, there have been few research reports on the synthesis of enzyme–AuNCs using the “top–down” method. Therefore, the dynamics of nanocluster formation during the use of the top–down approach also require further investigation for a better understanding of the role of enzyme ligands in the etching process.

In contrast to top–down methods, the “bottom–up” approach forms AuNCs through the reduction of Au atoms [33]. Many researchers have opted to reduce Au atoms to synthesize enzyme–AuNCs because producing nanoscale structures and materials using top–down techniques is challenging, as core etching is a harsh chemical process in which most proteins lose most of their structure, often resulting in a wider size distribution of the resulting particles [34]. The bottom–up approach provides the possibility of creating hierarchical and ordered nanostructures by taking advantage of the physicochemical interactions and self-assembly of nanoscale building blocks, which play very important roles in the final creation of nanomaterials with the desired functions and properties [35]. Additionally, the bottom–up method does not require harsh conditions to synthesize AuNCs, unlike the top–down process. This allows for the protein properties to be preserved after AuNC formation within the protein. Among the many bottom–up methods for synthesizing protein-templated AuNCs, the biomineralization of proteins is the most widely used method as a simple, one-pot, and green synthetic route [36]. In a typical synthesizing process, aqueous HAuCl_4_ solution is added to a protein (enzyme) solution under vigorous stirring; NaOH solution is then added, and the mixture is incubated at a given temperature for about 12 h [37]. During the reaction, NaOH works as a reducing agent, reducing Au ions into AuNCs. The AuNCs are thiol protected via the cysteine (Cys) residues in the protein and exhibit stability in solution, forming clusters [38]. Along with Cys residues, tyrosine (Tyr) residues can also be used to reduce/stabilize Au(III) through their phenolic groups [39]. By adjusting the reaction pH above the pKa of Tyr, NaOH also aids the reaction by increasing the pH and, thus, the reaction capability [40]. During the reduction reaction, the color of the solution changes, and the enzyme–AuNCs are successfully synthesized. Enzyme–AuNCs are verified as the final product by accounting for their specific properties, with their fluorescence typically measured for this purpose. In addition, their formation can be verified through more specific investigations using transmission electron microscopy (TEM) images, recording sizes under 2 nm. Although “bottom–up” methods are easier to use in the synthesis of enzyme–AuNCs than the “top–down” method, there are some key factors that influence the formation of AuNCs in enzymes using the bottom–up method [41]. For example, the pH can affect the reduction rate and final cluster size. For instance, Au clusters with 25 atoms (Au_25_) often grow at a pH of 11, while at a pH of 7–8, AuNCs with fewer than eight atoms grow (Au_8<_). The temperature can influence the reaction kinetics and protein conformation. In addition, different types of proteins (including enzymes) yield AuNCs with varying properties due to their unique structures and amino acid compositions. The gold-to-protein ratio also impacts the size and fluorescence properties of the resulting AuNCs. Therefore, to successfully synthesize the desired enzyme–AuNCs, a protocol must be developed with these key factors in mind.

Next, we would like to introduce representative features of enzyme–AuNCs that possess both protein-stabilized AuNCs and enzymes due to the stabilization of Au ions by enzymes.

### 2.2. Fluorescence Properties of Enzyme–AuNCs

Enzyme–AuNCs exhibit unique fluorescence properties. The AuNCs possess discrete electronic energy levels, and their size regime (of less than 2 nm) is comparable to the Fermi wavelength of conduction electrons. Therefore, the surface plasmon band disappears, and size-dependent fluorescence can be observed [42]. Apell et al. explained that recombination associated with *d*-band excitation brings about these fluorescence properties. The absorbed photons promote electrons from the narrow *d*-band to the empty *sp*-band above the Fermi level [43]. Radiative recombination responsible for a visible–NIR emission occurs between the electron and the excited hole when certain carriers relax. Interestingly, we can find that the fluorescent colors of the enzyme–AuNC are not all the same and that the color may vary depending on the stabilizing protein used. As elucidated by Wu et al., surface ligands (-SRs) play a major role in improving fluorescence [44]. Fluorescence changes are transferred from the ligand to the metal nanocore through the Au–S bond. In addition, fluorescence appears by directly donating non-localized electrons of electron-rich atoms or ligand groups to the metal core. As a result, enzyme–AuNCs have different-colored fluorescence depending on the stabilizing ligand enzyme they contain. For example, horseradish peroxidase (HRP)–AuNCs emit red fluorescence, but glucose oxidase (GOx)–AuNCs emit yellowish-green fluorescence.

### 2.3. Electrical Properties of Enzyme–AuNCs

Enzyme–AuNCs exhibit unique electrical properties. These properties are primarily influenced by the interaction between the gold core and the stabilizing enzyme.

First, enzyme–AuNCs have a much-improved conductivity over that of proteins [45]. In enzyme (including protein)–AuNCs, AuNCs have been found to play a critical role as conductive holders and accumulators of redox-active centers at the surface of working electrodes [46]. The size regime of AuNCs, comparable to the Fermi wavelength of conduction electrons, governs their properties [47]. For this reason, protein–AuNCs show better conductivity than proteins alone [48,49]. Second, AuNCs have been reported to function as nanozymes with peroxidase-like properties. As reported in previous research, H_2_O_2_ can be adsorbed onto the surface of AuNCs through the O–O bond of H_2_O_2_, and it can be decomposed into dihydroxy radicals [50,51]. At the same time, the produced hydroxyl radicals are stabilized by the AuNCs through partial electron-exchange interactions, contributing to their catalytic ability. These peroxidase-like properties boost the catalytic activities of enzymes and accelerate the reactions of enzyme–AuNCs. Third, the enzymatic properties of enzyme–AuNCs facilitate the catalysis of specific targets. Enzymes are proteins that act as biological catalysts by accelerating chemical reactions [52,53]. The molecules that an enzyme can react with are called substrates, and the enzyme converts the substrate into another molecule known as the product. Due to their selectivity in accelerating chemical reactions, enzyme–AuNCs have shown strong catalytic reactivity and selectivity compared to non-enzyme–AuNCs.

There are several factors that influence the electrical properties of enzyme–AuNCs, such as the size of the AuNCs, surface modification, and protein structure [54]. The size of the AuNCs plays a crucial role in determining their enzyme-like activity and electrical properties. According to the size of AuNCs, for example, Au_5_NC or Au_13_NC, their electrical properties are going to be different. The surface chemistry of AuNCs, including the charge and functional groups in the enzyme, can significantly impact their catalytic and electrical properties. Also, the conformation and structural integrity of the stabilizing protein or enzyme affect the properties of the AuNCs.

By harnessing the characteristics of both AuNCs and enzymes, enzyme–AuNCs have good optical properties and conductivity and permit rapid and selective enzyme reactions, confirming that they are very good candidate materials for applications in enzyme-based biosensors. The following sections introduce the advantages of enzyme–AuNCs in optical and electrochemical biosensors and present various types of enzyme–AuNCs that are applied to advanced biosensors.

## 3. Advanced Optical and Electrochemical Biosensors Using Various Types of Enzyme–AuNCs

In the field of biosensing, many researchers have been mainly choosing optical and electrochemical phenomena as sensing methods. Optical phenomena, including colorimetry, fluorescence, and surface-enhanced Raman spectroscopy, have many advantages in terms of their applications within biosensing methods due to their relatively cheap analysis and instrumentation and the ease of detection [55,56]. For these reasons, optical biosensing technology is one of the most preferred techniques for use in biosensors. Biosensors are integrated receptor–transducer devices, and the receptor part encompasses biological recognition elements such as nucleic acids, peptides, antibodies, and enzymes [57]. In optical biosensors, a series of processes allows the receptor to recognize the target analyte and convert this interaction into an optical signal [58]. Therefore, the selection of the materials that convert the target into the signal becomes very important. Enzyme–AuNCs are highly advantageous, as they function as both signal transducers and signal generators simultaneously, demonstrating unique optical properties, such as the catalytic properties of the enzymes and the autofluorescence of the AuNCs. Specifically, when an enzyme interacts with the target substrate, a phenomenon such as the aggregation of the AuNCs occurs, resulting in a change in the signal, such as fluorescence quenching. Thanks to this, enzyme–AuNCs can be used to detect targets without the need for additional signal generators, enabling easy detection with the minimum process.

Given the signal transduction–generation properties of enzyme–AuNCs, they are good candidate materials within electrochemical sensing technology. Electrochemical sensing technology has been applied to many biosensing-related fields due to its outstanding properties, such as its rapidity, high sensitivity, and simple detection methods [59,60]. Among the options, enzyme-based electrochemical biosensing has been a widely used method due to its high selectivity, predicated on a highly selective enzyme–substrate reaction [61]. In enzyme-based electrochemical sensing technology, effective electrical transfer between the redox center of the enzyme and the electrode is key to achieving excellent electrochemical detection [62]. However, there are also some disadvantages to using enzymes in electrochemical systems. For high sensitivity, large amounts of the enzyme need to be immobilized on the surface of the electrode. However, enzymes have a low conductivity, hindering the electron transfer in electrochemical biosensors [63]. As a result, the amount of the enzyme must be increased to amplify the signal, but increasing the amount of the enzyme also paradoxically leads to the signal decreasing. Enzyme–AuNCs offer potential solutions to this paradox due to their excellent electrochemical properties, such as high conductivity, and unique molecular redox properties. In the next section, we will introduce various types of enzyme–AuNC-based biosensors, focusing on their optical and electrochemical properties in particular. 

### 3.1. HRP–AuNC-Based Biosensors

HRP is a heme-containing enzyme extensively utilized in biochemistry, molecular biology, and biotechnology due to its catalytic efficiency and versatility. Derived from the root of the horseradish plant (*Armoracia rusticana*), HRP has a molecular weight of approximately 40 kDa [64]. This enzyme catalyzes the oxidation of a wide range of substrates using hydrogen peroxide (H₂O₂) as the oxidizing agent, following the reaction

$$H_{2}O_{2}+2AH_{2}\overset{HRP}{\to }2H_{2}O+2AH•$$

**Figure 2:**
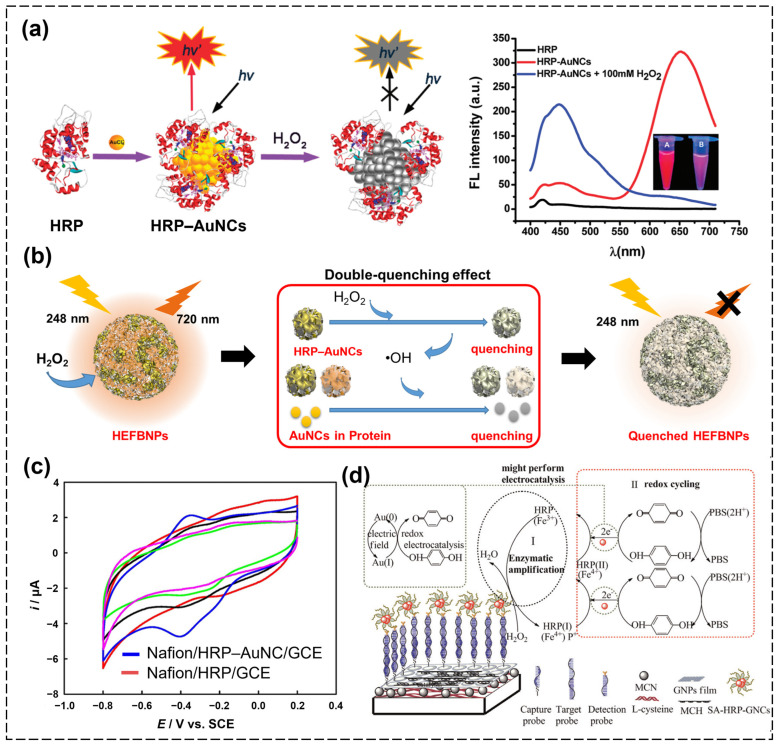
(**a**) Schematic illustration of the synthesis of HRP–AuNCs and their quenching of the fluorescence from H_2_O_2_ molecules, and inset graph showed the quenching of HRP–AuNCs at 650 nm in presence of H_2_O_2_ (red: HRP–AuNCs without H_2_O_2_; blue: HRP–AuNCs with 100 mM H_2_O_2_ [66]). (**b**) Schematic illustration of multi-HRP–AuNC−nanoparticle-based highly sensitive fluorescence detection of H_2_O_2_ [68]. (**c**) Cyclic voltammetry analysis of H_2_O_2_ using a bare glassy carbon electrode (GCE) (red curve), Nafion/HRP/GCE (black curve), Nafion/HRP–NaAuCl_4_/GCE (green curve), Nafion/HRP/Au plate/GCE (pink curve), and Nafion/HRP–AuNCs/GCE (blue curve) [73]. (**d**) The enzymatic amplification and redox cycling mechanism of an HRP–AuNC-probe-based electrochemical biosensor for the detection of a target DNA sequence [75].

HRP operates optimally at a near-neutral pH of around 7.0 and within a temperature range of 30 °C to 40 °C. In particular, due to the presence of Cys residues in HRP, this enzyme was posed as a candidate for stabilizing AuNCs via Au–S bonding [65]. HRP–AuNCs were sustainably synthesized for the first time in 2011 by Wen et al. under physiological conditions using the bottom–up method. The synthesized HRP–AuNCs were found to not only maintain the catalytic function of HRP as an enzyme but also exhibit the fluorescence properties of the AuNCs [66]. Consequently, the dual function of the fluorescence of AuNC and the catalytic ability of the enzyme shell allows for the use of HRP–AuNCs as a material capable of target recognition and signal generation simultaneously. In the normal state, the HRP–AuNCs emitted red fluorescence (λ_em,max_ = 650 nm), but when they were catalyzed with H_2_O_2_, their fluorescence was quantitatively quenched (Figure 2a). Prior to this study, bovine serum albumin (BSA)–AuNCs were widely used for fluorescence detection of H_2_O_2_, but HRP–AuNCs showed more sensitive signals than BSA–AuNCs. This was a result of the catalytic activity of HRP, with the Au–S bonding between the HRP scaffolds and the encapsulated AuNCs oxidizing to form a disulfide product in the presence of reactive oxygen species (ROS). As a result, the AuNCs with HRP were prone to aggregate more than BSA–AuNCs, leading to the effective quenching of fluorescence. To prove that the catalytic activity of HRP plays a major role in H_2_O_2_-induced quenching, Wen et al. conducted a comparison experiment by employing BSA–AuNCs as a control. The addition of 100 μM H_2_O_2_ induced a significant decrease in the two peaks (650 and 450 nm) ratio (denoted as I_650_/I_450_) for HRP–AuNCs. On the contrary, the existence of 100 μM H_2_O_2_ caused a neglectable variation in the peak ratio (I_670_/I_450_) in the case of BSA–AuNCs. This excellent performance, with the fluorescence of HRP–AuNCs being quenched by H_2_O_2_, made it a good candidate to apply to the fluorescence strategy in microdroplet biosensors for the sensitive detection of H_2_O_2_ secreted by various cell lines. Shen et al. suggested a microfluidic approach using single-cell encapsulated droplets in combination with HRP–AuNCs for the detection of H_2_O_2_ [67]. They demonstrated a high sensitivity (limit of detection, LOD = 1.0 nM), providing sufficient sensitivity for the single-cell released H_2_O_2_ level, offering a useful tool for studying cell-to-cell differences in H_2_O_2_ secretion at the single-cell level. The results from microdroplet-based methods can achieve a detection limit of nearly three orders of magnitude better than the traditional assays. 

While many papers have combined AuNCs with H_2_O_2_-related enzymes for detection purposes, the sensitivity achieved has not been sufficient to detect very low concentrations of H_2_O_2_. To increase the sensitivity of H_2_O_2_ detection, Lee et al. proposed the HRP–AuNC-encapsulated fluorescent bio-nanoparticles (HEFBNPs) composed of HRP–AuNCs and BSA–AuNCs (Figure 2b) [68]. The HEFBNPs showed high fluorescence quenching at a low concentration of H_2_O_2_ compared to that when solely using HRP–AuNCs due to the synergistic effect of the different, continuous two-step fluorescence quenching mechanisms associated with the HRP–AuNCs and BSA–AuNCs. When the HRP–AuNCs enzymatically reacted with H_2_O_2_, they produced hydroxyl radicals (•OH), and, at the same time, the HRP–AuNCs were quenched due to their agglomeration. Continuously, •OH reacted with the AuNCs with its strong oxidative power, decreasing the intensity of the fluorescence. In addition, the proximity of the two types of protein–AuNCs was able to decrease the loss of intermediate (•OH), which also increased the rate at which the AuNCs were quenched. As a result, a wide range of concentrations of H_2_O_2_ of up to 0.5 nM was detected with good linearity and selectivity. In addition to the fluorescence properties of HRP–AuNCs, the properties of AuNCs that increase the catalytic activity of HRP can be applied to the high-sensitivity enzyme-linked immunosorbent assay (ELISA). Dai et al. suggested an HRP–AuNC-based capture-detection platform for the detection of anti-PDL1 autoantibodies (anti-PDL1 AAbs) [69]. With the nano capture probe (MB peptide) and the detection nanoprobe, anti-IgG-HRP-AuNCs, the platform achieved remarkably sensitive and specific detection of anti-PDL1 Aab. Compared to the conventional ELISA detection system using an the HRP probe, the innovative nanoplatform not only reduced detection time (12h to 50 min) but also exhibited significantly greater specificity in diagnosing early-stage lung cancer (suggested platform: positive rate of 93.7% with a specificity of 64.6%; traditional ELISA: positive rate of 84.8% and a specificity of just 54.4%).

In electrochemical biosensors, H_2_O_2_ is widely used as a target given that it can be either oxidized or reduced directly on ordinary solid electrodes [70,71]. Although H_2_O_2_ can be detected by utilizing the fluorescence properties of HRP–AuNCs, measurements of this kind are difficult for the real-time and dynamic detection of H_2_O_2_ in vivo because their reactions are irreversible, and they can only reflect transient concentrations. On the other hand, electrochemical techniques that measure the electron transfer between an electrode and H_2_O_2_ are more suitable for continuous H_2_O_2_ measurements and monitoring [72]. However, these processes need to solve the limitation of analytical applications, which come from slow electrode kinetics and high overpotential, inducing poor sensing performance. Also, they may suffer from significant interference from the other electroactive species present in real samples, such as ascorbate, urate, and nitrates. On this basis, HRP–AuNCs are primed for use in H_2_O_2_-detecting electrochemical biosensors given their high specificity, enhanced enzymatic reactions, and good conductivity. He et al. first reported the development of an HRP–AuNC-based electrochemical biosensor for H_2_O_2_ analysis [73], discerning that an HRP-AuNC-based electrode showed superior electrochemical properties compared to the HRP-based electrode that was not decorated with AuNCs (Figure 2c). As shown in Figure 2c, no obvious redox peaks were seen for the bare GCE (red curve), and the peaks in the cyclic voltammogram were also very weak for Nafion/HRP/GCE (black curve), Nafion/HRP–NaAuCl_4_/GCE (green curve), and Nafion/HRP/Au plate/GCE (pink curve). In vivid contrast, a pair of stable and well-defined redox peaks was seen for Nafion/HRP–AuNCs/GCE (blue curve), with an apparent formal potential (E_0_′) of −389 mV and a peak-to-peak separation (ΔE_p_) of 42 mV, indicating a quasi-reversible redox process for the HRP. Moreover, they calculated the electron transfer rate constant (k_s_) and showed that the k_s_ value of Nafion/HRP-AuNCs/GCE (3.19 s^−1^) was larger than those of Nafion/HRP/GCE (2.34 s^−1^), Nafion/HRP-NaAuCl4/GCE (1.75 s^−1^), and Nafion/HRP/Au_plate_/GCE (0.70 s^−1^) and is also larger than that of HRP/Au colloid film (1.84 s^−1^). The explanation for this strong electrochemical performance is that the AuNCs aid effective electrical communication between the redox center of the enzyme and the electrode-like electric wire. In addition, the HRP–AuNC-based electrode exhibited a pair of well-defined cyclic voltametric peaks and high electrocatalytic activity for the reduction of both O_2_ and H_2_O_2_ due to the synergistic effects of the HRP and the nearby AuNCs. As a result of the enhancement in the electrochemical properties provided by the HRP–AuNCs, H_2_O_2_ was detectable with an LOD of 0.99 μM, which shows higher sensitivity compared to HRP with an LOD of 1.2 μM. In addition, the biosensor showed good stability with only a 13% current decrease after 1 month of storage. Similarly, Ren et al. developed an HRP–AuNC-based electrochemical biosensor for the sensitive detection of H_2_O_2_ [74]. Instead of Nafion/GCE, they applied multiwalled carbon nanotubes (MWCNTs) onto the surface of carbon fiber ultramicroelectrodes (CFUMEs) with HRP–AuNCs (HRP–AuNCs/MWCNTs/CFUMEs). Due to the enhanced surface charge and enlarged surface area provided by the MWCNTs, the fabricated electrode showed enhanced sensitivity compared with Nafion/HRP/GCE, with an LOD of 443 nM and sensitivity of 3.0 × 10^−4^ A/M.

In electrochemical biosensors, HRP–AuNCs can also be applied for the direct detection of H_2_O_2_ due to their high conductivity; they also constitute a good electrochemical probe given the electrocatalytical reactions of the Au (I) and Au (0) surface sites on the nanoclusters, which enhance the enzymatic reactions with H_2_O_2_. For example, Zhou et al. developed an electrochemical DNA detection platform using Streptavidin–HRP-stabilized AuNCs (SA–HRP–AuNCs) as the electrochemical probe and a mesoporous carbon nitride (MCN)/DNA-conjugated, AuNP-modified carbon electrode (Figure 2d) [75]. When the target DNA was captured by the DNA probe on the modified electrode, the SA–HRP–AuNCs, as the DNA detection probe, could immobilize the DNA on the electrode through hybridization of the DNA. In this system, the SA–HRP–AuNCs performed an important role in signal amplification. Since the SA–HRP–AuNCs worked as electrocatalysts for the substrate (H_2_O_2_) as well as electron transducers, an enhanced electrochemical signal was observed compared to the signal produced using the bare SA–HRP. Due to their excellent enzymatic properties, the HRP–AuNCs could detect a range of target DNA from 100 aM to 10 nM with an LOD of 8.0 aM under the optimized conditions. Compared to an enzyme (SA–HRP)-labeled and CNT-based sensor and other enzyme-based electrochemical DNA sensors, the HRP–AuNC-based biosensor exhibited improved analytical performances.

### 3.2. Catalase–AuNC-Based Biosensors

Catalase (CAT) is an enzyme with four heme groups per tetramer [76]. It has a molecular weight ranging from approximately 220 to 350 kDa and exhibits optimal activity at a pH between 7 and 8 and at temperatures ranging from 37 °C to 40 °C [77]. It contains twenty Tyr residues and four Cys, with the Cys stabilizing the metal core and acting as ligands.

CAT has similar properties to HRP—for example, it can catalyze the decomposition of H_2_O_2_—but it also differs in some of its properties. In terms of the enzymes’ structures, Tyr constitutes the proximal heme ligand in CAT, while histidine is the proximal heme ligand in HRP. From the point of view of enzymatic reactions, CAT has a lower energy barrier (6 kcal/mol) compared to HRP (18.7 kcal/mol) in the degradation of H_2_O_2_. Also, CAT has two pathways for H_2_O_2_ degradation (while HRP has one) and has dry active sites that do not require water. Thus, CAT is generally considered more efficient for the degradation of H_2_O_2_ than peroxidase. The main differences in the mechanisms of H_2_O_2_ degradation between both enzymes are as follows:

$$2H_{2}O_{2}\overset{Catalse}{\to }2H_{2}O+O_{2}\;(2\;\mathrm{electron}\;\mathrm{transfers})$$

$$H_{2}O_{2}+2AH_{2}\overset{Peroxidase}{\to }2H_{2}O+2AH•\;(1\;\mathrm{electron}\;\mathrm{transfer})$$

**Figure 3:**
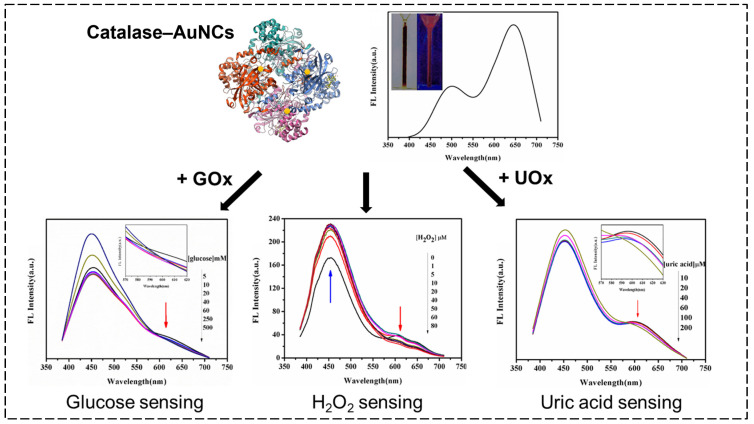
The emission spectra of CAT–AuNCs (λ_ex_ = 365 nm); the inset figure shows photographs of CAT–AuNCs under room light and a UV (365 nm) lamp; their application in H_2_O_2_, glucose, and uric acid sensing [78].

These characteristic differences result in CAT being more specialized for H_2_O_2_ decomposition. In addition, in terms of the stabilization of AuNCs, the reducing properties of the Tyr residues in CAT enable the reduction of Au ions under highly alkaline conditions, making it an attractive option for stabilizing AuNCs. In 2018, Meng et al. synthesized the first CAT-stabilized AuNCs (CAT–AuNCs) under alkaline conditions [78]. These CAT–AuNCs, with CAT serving as both a reducing and stabilizing agent in their synthesis, exhibited dual emission peaks at 490 nm and 650 nm (Figure 3). Upon detecting H₂O₂, a 37 nm blue shift in the entire emission wavelength was observed, attributed to the disruption of Au–S bonds and conformational changes in the CAT molecules, leading to fluorescence quenching. In further detail, the RS–Au (R = CH_3_(CH_2_)_n_) bonds were oxidized into RS–SR and even RSO_3_H by H_2_O_2_. In the presence of H_2_O_2_, the quenching reaction of the CAT–AuNCs was as follows:

$$Low\;concentration\;of\;H_{2}O_{2}:2RS–Au\to RSSR+2Au$$

$$High\;concentration\;of\;H_{2}O_{2}:RS–Au\to RSO_{3}H+HO–Au$$

These reactions lead to rapid changes in the structure of the AuNCs and effective fluorescence quenching of the CAT–AuNCs. As a result, H₂O₂ was detectable within a linear range of 10–80 μM with an LOD of 25 nM. Additionally, the CAT–AuNCs were employed to detect glucose and uric acid utilizing GOx and urate oxidase (UOx), respectively:

$$Glucose+O_{2}+H_{2}O\overset{GOx}{\to }Gluconic\;acid+H_{2}O_{2}$$

$$Uric\;acid+O_{2}+H_{2}O\overset{UOx}{\to }Allantoin+H_{2}O_{2}+CO_{2}$$

$$CAT–AuNCs\left(F\right)+H_{2}O_{2}\to CAT–AuNCs\left(Q\right)+H_{2}O+{\frac{1}{2}O}_{2}$$

(F: Fluorescence−emitting form, Q: Fluorescence−quenching form)

These reactions produce H₂O₂, which quenches the fluorescence of the CAT–AuNCs. Consequently, using the CAT–AuNCs with GOx or UOx, glucose could be detected within a range of 5–500 μM with an LOD of 3 mM, while uric acid could be detected within a range of 10–200 μM with an LOD of 40 nM. From the comparison of the efficiency of the CAT–AuNCs with other materials, such as AuNPs, BSA–AuNCs, and carbon quantum dots (CQDs), the CAT–AuNC-based sensors showed good results. The author claimed that the results could be attributed to both the activity of the CAT and dual-emission fluorescence characteristics of the CAT–AuNCs.

### 3.3. GOx–AuNC-Based Biosensors

**Figure 4:**
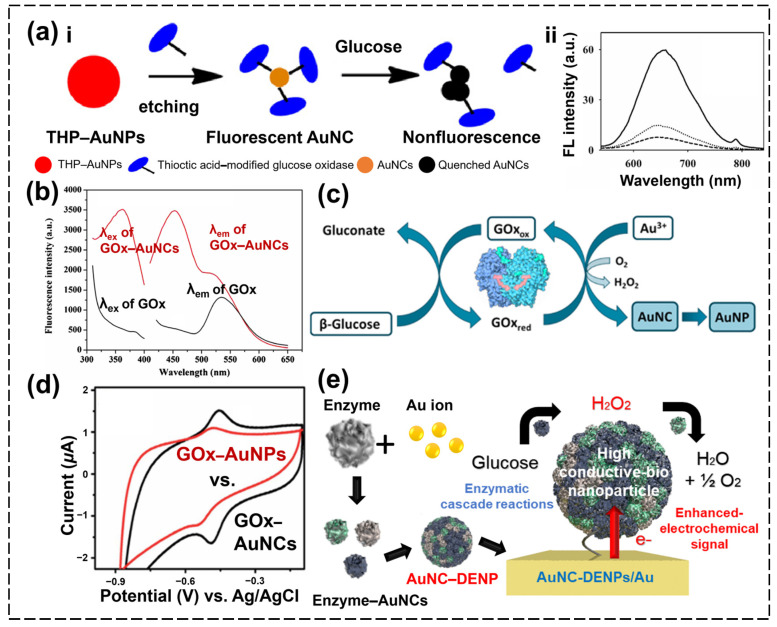
(**ai**) Schematic illustration of “top–down” synthesis and fluorescence quenching of GOx–AuNCs and (**aii**) fluorescence spectrum of GOx–AuNCs according to the addition of glucose (glucose concentration: 0 (solid), 1.0 × 10^−4^ (dotted), and 1.0 × 10^−3^ M (long dash)) [82]. (**b**) Fluorescence excitation and emission spectra of bare GOx and GOx–AuNCs [83]. (**c**) Schematic illustration of the formation of AuNCs and AuNPs from the enzymatic oxidation of glucose catalyzed by glucose oxidase (subscripts “ox” and “red” refer to the oxidized and reduced forms of GOx, respectively, in the presence of Au(III)) [84]. (**d**) Comparison of cyclic voltammetry results between GOx–AuNCs (black) and GOx–AuNPs (red) [87]. (**e**) Schematic illustration of a highly sensitive glucose electrochemical sensor using GOx/HRP–AuNC–based nanoparticles [90].

GOx is an enzyme with distinct biochemical and structural properties, making it valuable for various scientific and industrial applications. Structurally, GOx is a dimeric protein with a molecular weight of approximately 160 kDa, with each monomer representing around 80 kDa [79]. The enzyme consists of two identical polypeptide chains that comprise around 583 amino acid residues each, and each monomer binds one molecule of flavin adenine dinucleotide (FAD), which is crucial to the enzyme’s catalytic activity. As a homodimer, the two identical subunits in GOx confer its stability and function. Biochemically, GOx exhibits high specificity for β-D-glucose (glucose), catalyzing its oxidation into H_2_O_2_ and D-glucono-δ-lactone (which is further converted into gluconic acid) [80]. Structurally, the presence of Cys residues (thiol compounds) in its molecular structure makes GOx a potential template for the direct synthesis of fluorescent AuNCs [81]. GOx–AuNCs were first synthesized by Xia et al. by etching AuNPs using thioctic acid-modified GOx according to a top–down method (Figure 4(ai)) [82]. Due to the significant fragmentation energies, once the thiol capping agent is absorbed onto the particles’ surface, AuNPs are prone to dissociating into smaller forms and forming Au–thiolate clusters, enabling the formation of GOx–AuNCs. The GOx–AuNCs fabricated are then usable as enzymes and fluorescence signal probes (λ_em,max_ = 650 nm, emitting red fluorescence). The main quenching mechanism is as follows:

$$Glucose+O_{2}+H_{2}O\overset{GOx–AuNCs}{\to }Gluconic\;acid+H_{2}O_{2}$$

$$GOx–AuNCs\left(F\right)+H_{2}O_{2}\overset{Catalase–likeactivity}{\to }GOx–AuNCs(Q)+H_{2}O+{\frac{1}{2}O}_{2}$$

(F: Fluorescence−emitting form, Q: Fluorescence−quenching form)

As the GOx–AuNCs catalyzed the reaction of glucose and dissolved O_2_ to produce H_2_O_2_, the AuNCs were quenched by H_2_O_2_ (Figure 4(aii)). By analyzing the intensity at which the fluorescence of the GOx–AuNCs was quenched, this research team were able to sense glucose within the linear range of 2.0–140 μM with an LOD of 0.7 μM. In contrast, Cui et al. synthesized GOx–AuNCs using the bottom–up method of a “one-pot” biomineralization approach, with the AuNCs formed through the reduction of Au ions [83]. The GOx–AuNCs fabricated emitted blue fluorescence (λ_ex,max_ = 360 nm, λ_em,max_ = 450 nm), differing from the results of Xia’s group (Figure 4b). The reason for this difference is that the structure or size of the AuNCs varies depending on the method with which the clusters are formed, and on this basis, the fluorescence characteristics may vary. They applied GOx–AuNCs for detecting H_2_O_2_. Unlike other AuNCs, such as BSA-stabilized AuNCs and HRP-stabilized AuNCs, Cui et al.’s GOx–AuNCs do not observably undergo fluorescence quenching in the presence of H_2_O_2_. However, the addition of a Fenton reaction, which converts H_2_O_2_ into OH•, facilitated the quenching of the fluorescence of the GOx–AuNCs by H_2_O_2_, and this effect was applicable to determining the H_2_O_2_ levels in the 0.5 to 10 μM concentration range. Also, the sensitivity of this method was higher than that of previous reported methods and comparable to that of the chemiluminescence method and SERS using bare HRP, polymers, and AuNPs. Uniquely, the intensity of the fluorescence of the GOx–AuNCs was restored in the presence of antioxidants. The research team was able to exploit this property to monitor the H_2_O_2_ scavenging activity of certain antioxidants, assessing the efficiencies of ascorbic acid and tartaric acid. Meanwhile, Camacho-Aguayo et al. also synthesized GOx–AuNCs using a bottom–up method [84]. Interestingly, they discerned that when the oxidation of glucose by GOx was carried out in the presence of Au(III), gold nanomaterials (AuNCs and AuNPs) were observed to form in situ (Figure 4c). In particular, they determined that the ratio of the AuNCs and AuNPs to GOx varied depending on the experimental conditions during the reaction with glucose and GOx. Moreover, they also found that the intensity of the fluorescence and the UV absorbance of the AuNCs and AuNPs, respectively, were determined by the glucose concentration, which affected the formation of these gold nanomaterials. In summary, when working at a pH of 6, only AuNCs with λ_em,max_ = 420 nm (λ_ex,max_ = 335 nm) were obtained, and the intensity of their fluorescence increased with the concentration of glucose (according to a linear relationship from 6.0 × 10^−5^ M to 1.5 × 10^−3^ M glucose). However, when the enzymatic reaction was performed at a pH of 8, AuNPs (with an absorption peak at 580 nm) were also obtained, and their intensity increased with glucose concentration (according to a linear relationship from 5.5 × 10^−4^ M to 2.0 × 10^−3^ M glucose). They applied these findings to determine the concentration of glucose in both orange juice and a human plasma sample and statistically compared them with the traditional analysis method using the HRP/GOx/TMB method at a confidence interval of 95%, and no significant differences were found, which mean that the proposed method’s accuracy was high.

GOx is a glycoprotein with a rigid structure that contains FAD, which takes part in two-electron, two-proton direct electron transfer in redox reactions [85]. Although GOx can be investigated in terms of its redox activity as a glucose catalyst using electrochemical methods, this is hard to detect on the basis of the direct electron transfer between GOx and the electrode. This is because the active center, FAD, is buried deep inside the protein [86]. However, GOx–AuNCs offer solutions to this problem. Muthurasu et al. demonstrated that GOx–fluorescent AuNPs (which were the same as GOx–AuNCs) emitted an intense yellowish-green color upon excitation at 365 nm [87]. The research team applied these GOx–AuNCs to a GCE, finding that the electrode exhibited superior electrochemical properties compared to a GOx–AuNP-based electrode and good sensitivity for glucose (Figure 4d). This result showed that GOx–AuNCs are more adjustable for electrochemical biosensing than bare GOx. This difference stems from the fact that the AuNCs facilitate direct electron transfer between glucose oxidation and electrode. In addition, the GOx–AuNCs showed lower K_m_ values (Michaelis constant) for the electrochemical detection of glucose, meaning that the AuNCs were able to enhance the efficiency of the enzymatic catalysis of GOx (K_m_: GOx–AuNCs = 0.05, GOx–AuNCs = 0.334, and GOx–Au CNT = 14.9). Recently, efforts have been made to increase the sensitivity of electrochemical sensors for glucose that use AuNCs by incorporating nano-enzyme technology. Conductivity is generally an important factor for sensitive detection using enzyme–AuNC-based electrochemical biosensors, but the speed of the enzyme reactions is also an important factor [88]. An easy way to enhance the catalytic activity in electrochemical biosensors is to immobilize large amounts of the enzyme on the electrode and increase the diffusion rate of the enzyme into the substrate or decrease the loss of electron energy during diffusion [89]. To increase the sensitivity of the detection of glucose by increasing the enzyme activity while maintaining good conductivity in the electrode, Lee et al. proposed the use of AuNC–embedded dual-enzyme nanoparticles (AuNC–DENPs) with a large number of GOx–AuNCs and HRP–AuNCs encapsulated into one particle (Figure 4e) [90]. The AuNC-DENPs combined the enzymatic reactions of both GOx–AuNCs and HRP–AuNCs, meaning that glucose was oxidized, and H_2_O_2_ was reduced continuously. In these reactions, the proximity of the two enzymes within a single nanoparticle reduced the loss of the intermediate (H_2_O_2_) and enhanced the catalytic activity of AuNC–DENPs for glucose. In addition, given that the AuNC–DENPs were composed of AuNCs, they showed good conductivity. Due to the high loading of the enzyme into each nanoparticle, the reduced loss of the intermediate, and their good conductivity, these AuNC–DENPs were applied in a highly sensitive electrochemical biosensor for the detection of glucose by conjugating them onto the surface of a gold electrode. As a result, the AuNC–DENP/Au electrode-based electrochemical sensor showed very high sensitivity and selectivity for glucose, with a detection range from 5 to 320 nM (LOD = 2.58 nM) and good stability, maintaining 70% performance while in a repetitive-use environment for 30 days. In addition, the AuNC–DNEP-based biosensor showed good sensitivity compared to other GOx-based nanoprobe-based biosensors (sensitivity: GOx/AuNP = 4.31, GOx/Au/carbon/TiO_2_ = 29.76, GOx/AuNP/BSA/Fe_3_O_4_ = 115.3, and AuNC-DENP = 18,944 μA/mM cm^2^).

### 3.4. Template Enzyme–AuNC-Based Biosensors

In addition to using specific enzymatic reactions to target analytes, enzymes have received tremendous attention as stable templates for AuNC formation. Enzymes are suitable as stabilizing templates for AuNCs due to properties such as their high molecular weight, various amino acid compositions, and unique physical structures [91].

For example, among various types of enzymes, lysozyme (Lys) has been spotlighted as a stabilizer for AuNCs in many studies. Lys plays a crucial role in stabilizing AuNCs for several key reasons. The protein structure of Lys, particularly its α-helix domain, is well suited to encapsulating and stabilizing AuNCs [92]. Specific amino acid residues within Lys, especially Tyr-20 (Tyr20), are critical in facilitating the formation of AuNCs by promoting the reduction of Au ions [93]. In addition, all of the positively charged residues that constitute Lys’s primary structure are located on the protein’s surface, making it highly positively charged; thus, the negatively charged chloride ions produced as a result of the reduction of gold salt are prone to binding to the protein’s surface, inducing the attachment of excess gold salt as well. Overall, Lys effectively encapsulates AuNCs, protecting them from the external environment and enhancing their stability [94]. These characteristics collectively make Lys an effective agent for the synthesis and stabilization of AuNCs, resulting in the formation of stable nanoclusters with diverse biological and medical applications. According to these properties, Chen et al. were able to synthesize Lys–Au_8_NCs at a pH of 3 [95]. Among the various types of Lys, they selected Lys VI as the template, which was relatively effective in protecting the Au_8_NCs from polar solvents relative to other proteins. To verify their hypothesis, they compared the stability of BSA–AuNCs and the Lys VI–Au_8_NCs in the presence of formic acid, which can trigger AuNC agglomeration. As a result, they confirmed that Lys VI–AuNCs are more stable than those prepared using BSA. This research group applied the Lys VI–Au_8_NCs for the detection of GSH, which plays a pivotal role in cellular detoxification and may be a critical factor in brain damage, HIV expression, and cancer therapy. According to their experiment results, GSH can etch Au_n_NCs (*n* < 25, *n*= number of gold atoms) but not Au_25_NCs. Upon adding GSH to the Lys VI–Au_8_NCs, Au_8_ clusters were indeed etched into GSH–Au^+^ complexes by the GSH, and the fluorescence was quenched. On the basis of this phenomenon, the group was able to detect GSH in the linear range of 0.06 to 10 μM with an LOD of 20 nM, which is much lower than the normal level (0.5 to 10 mM), in erythrocytes.

**Figure 5:**
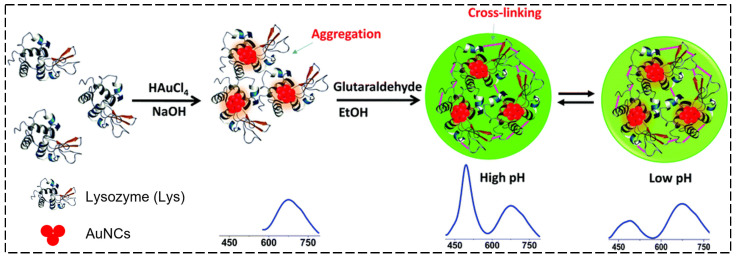
Schematic illustration of the synthesis of dual-emission LysNP–AuNCs for ratiometric pH sensing based on a combination of two synthetic strategies, including the Lys-mediated formation of AuNCs and glutaraldehyde-induced cross-linking between Lys and AuNCs [96].

Taking advantage of Lys–AuNCs, Chandirasekar et al. suggested and then developed lysozyme nanoparticle-encapsulated AuNCs (LysNP–AuNCs) for use as a ratiometric pH sensor based on the covalent cross-linking of the Lys–AuNCs (Figure 5) [96]. The AuNCs in the LysNP–AuNCs were found to be strongly embedded into the LysNPs, protecting the fluorescence of the AuNCs from photobleaching and under high salt concentrations more adequately than Lys–AuNCs, which enabled specific pH sensing. They determined the LysNPs to be pH responsive given that the green fluorescence of the LysNPs gradually decreased as the pH was changed from 9.5 to 7.5. This is because the α-helical structure of the LysNPs was gradually changed upon increasing the pH of the solution. In contrast, the red fluorescence of the AuNCs was insensitive to changes in the pH. This difference occurs in the variation of the two fluorescence emissions, and when the green and red channel images were overlapped, the merged images appeared orange, yellow, and bright yellow at a pH of 7.0, 8.0, and 9.0, respectively. The pH-induced change in the fluorescence of the LysNPs enabled the use of LysNP–AuNCs to ratiometrically detect slight changes (to 0.2 pH units) in the pH range from 7.5 to 9.5. Additionally, the LysNP–AuNCs were well suited to probing enzymatic reaction-induced changes in the pH, as exemplified in the context of urease-mediated hydrolysis of urea. Moreover, the LysNP–AuNCs were applicable to the detection of changes in the pH inside HeLa cells and provided great potential for monitoring mitochondrial pH levels (pH = ~8.0) and intracellular changes in the pH after treatment with cancer drugs.

As another example, Li et al. proposed a method for synthesizing Lys-AuNCs using a one-step microwave-assisted method optimized via four main reaction parameters: the molar ratio of Au^3+^/Lys, the concentration of NaOH, the reaction temperature, and the reaction time [97]. Their Lys–AuNCs showed beneficial fluorescence properties and could sensitively detect folic acid molecules. Comparing the sensitivities of previously reported probes (LOD of BSA–AuNCs = 65 nM, BSA–Au/AgNCs = 0.47 nM, OVA–CuNCs = 0.18 μM), the Lys–AuNCs showed very good sensitivity, with an LOD of 0.19 nM for folic acid. In addition, hyaluronic acid (HA)–coated Lys–AuNCs remarkably targeted tumors that were attributed to the number of receptors expressed by the cells. With co-engineering with other materials, Lys-AuNCs could be applied in biomedical field [98]. Wang et al. suggested AuNCs co-engineered with Lys and curcumin (LC–AuNCs) that exhibit GOx–like activity and ROS-scavenging ability. LC–AuNCs enabled the depletion of excessive glucose without generating toxic H_2_O_2_, thereby significantly alleviating oxidative stress. They applied LC–AuNCs to a hydrogel system and showed exceptional properties in terms of anti-infection, antioxidant effects, glucose depletion, immunoregulation, and pro-regeneration in vivo. In addition, the intrinsic fluorescence of LC–AuNCs allows for the direct visualization of the wound-healing process.

**Figure 6:**
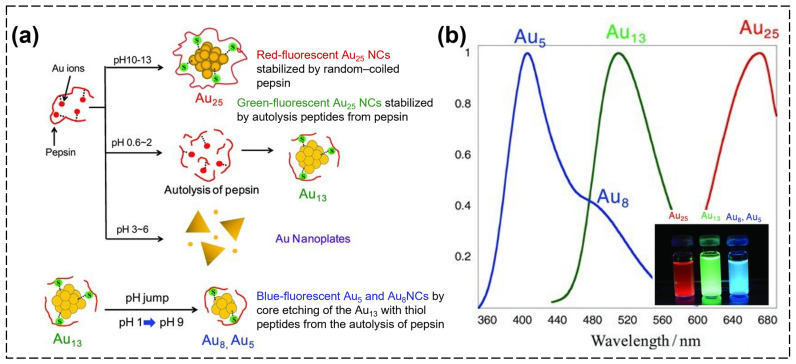
(**a**) Schematic illustration of the pH-dependent synthesis of Peps–AuNCs. (**b**) Fluorescence spectra of synthesized Peps–AuNCs with red (Au_25_ at a pH of 12), green (Au_13_ at a pH of 1), and blue fluorescence (Au_5_ and Au_8_ at a pH of 9). Inset image: Peps–AuNCs with red, green, and blue emissions under UV light [99].

Pepsin (Peps) is a gastric aspartic proteinase (34.5 kDa) that plays an integral role in the digestive process in vertebrates. It has unique properties in that it has high acidic amino acid and Tyr contents. Peps contains 18 Tyr, 6 tryptophans, 7 Cys, 71 dicarboxylic acids with 36 amides, and 35 free carboxyl groups. Unlike other enzymes, Peps can work in strong acidic conditions, such as stomach acid, and remain resistant to acids. In addition, under these harsh acidic conditions, the capacity for autolysis of Peps can be demonstrated. These properties make it possible for Peps–stabilized AuNCs (Peps–AuNCs) to be synthesized in acidic conditions. In 2011, for the first time, Kawasaki et al. adjusted the pH of a Peps solution to different values in order to synthesize various types of Peps–AuNCs (Figure 6a) [99]. Using Peps and by adjusting the pH, they were able to produce Peps–Au_13_NCs with green emissions (λ_ex_ = 330 nm, λ_em_ = 510 nm) at a pH of 1; Peps–Au_5_(Au_8_)NCs with blue emissions (λ_ex_ = 402 nm, λ_em_ = 480 nm) at a pH of 9; and Peps–Au_25_NC with red emissions (λ_ex_ = 360 nm, λ_em_ = 670 nm) at a pH of 12 (Figure 6b). In this research, they found that early-stage products of Peps hydrolysis with small internal spaces templated smaller Au_13_NCs at a low pH, while using denatured Peps with large internal spaces allowed them to form larger Au_25_NCs at an alkaline pH. Interestingly, Au_5_NCs and Au_8_NCs with blue fluorescence were only synthesizable from the Au_13_NCs through a jump in the pH from 1 to 9. That is, Au_13_NCs were required as the seed NCs in order to obtain AuNCs with blue fluorescence. Moreover, Peps–AuNCs could be applied as highly sensitive and selective sensors of Hg^2+^ according to fluorescence quenching given that BSA- and Lys-stabilized fluorescent Au_25_NCs are known as highly sensitive and selective sensors of Hg^2+^. As a result, this research group successfully detected Hg^2+^ using Peps–Au_25_NCs with red emissions within the linear range of 1–200 nM and with an LOD of 1 nM.

Table 1 provides a summary of the types of enzyme–AuNCs in play and their applications in various biosensors. Overall, enzyme–AuNCs are very attractive substances for the highly specific and sensitive detection of targets. Given their concurring excellent visual and electrochemical properties and enzymatic properties, further developments in using enzyme–AuNCs in various types of biosensors are expected.

## 4. Challenges and Specific Recommendations for Future Research Directions

### 4.1. Current Challenges of Enzyme–AuNCs in Application of Biosensors

Enzyme–AuNCs have emerged as promising materials for various applications, particularly in biomedicine and biosensing. However, they face several challenges that need to be addressed to fully realize their potential.

First, enzyme–AuNC-based biosensors should solve unintended reactions of signal development. Although enzyme–AuNCs have shown good specificity for their targets, certain metal ions, such as Hg^2+^ or Fe^2+/3+^, and ROS can affect the fluorescence intensity of the AuNCs [100,101,102]. This affects the performance of biosensors to detect specific target substances according to fluorescence changes in enzyme–AuNCs.

Second, they may limit the catalytic activity in neutral environments. One of the primary challenges is the restricted catalytic activity of enzyme-stabilized AuNCs at physiological pH levels. This limitation significantly constrains their potential applications, particularly in biomedical contexts [103]. For instance, the catalase-like activity of AuNCs is often substantially reduced in neutral environments, which is problematic for their use in biological systems.

Third, modifications to the AuNCs’ surface, such as the addition of coating molecules or stabilizing agents, can inadvertently block their active sites. This interference can potentially reduce or even inhibit their enzymatic activity. For example, in the experimental results of Griep et al., they showed that Au clusters being located close to the enzymatically active region of trypsin can prevent its activation [104]. Although the benefits of AuNCs may also benefit the properties of enzymes, they can also hinder enzymatic reactions. Therefore, not all enzymes are suitable for stabilizing ligands for AuNCs, nor are all synthesis protocols suitable. Therefore, it is also important to design the synthesis protocol of AuNCs so that it does not affect the enzyme working site when constructing the enzyme–AuNCs. Thus, it still requires a deeper understanding to maintain the desired functionality of the AuNCs while improving other properties like their stability or targeting capabilities.

Fourth, controlling the size, shape, and mono-dispersity of enzyme-stabilized AuNCs during synthesis remains a significant challenge [105]. The presence of enzymes can influence the reduction of kinetics and growth process of the nanoparticles, making it difficult to achieve consistent and reproducible results. This variability can lead to inconsistent performance across different batches of AuNCs.

Fifth, the enzyme–AuNCs developed thus far have been limited to several specific enzymes. As shown in this review, the use of enzymes to stabilize AuNCs has been limited to examples such as HRP, CAT, and GOx. These enzyme–AuNCs are used to selectively measure a target accordingly. In other words, specific enzyme–AuNCs must be developed for specific targets. Therefore, it is necessary to develop more diverse enzyme–AuNCs and develop new mechanisms to allow them to develop more diverse and scalable biosensors.

Sixth, the catalytic activity of enzyme-stabilized AuNCs is often highly dependent on the pH. For example, under acidic conditions, the catalase-like activity of AuNCs is significantly reduced. This pH sensitivity can be problematic when AuNCs are trapped in organelles such as endosomes (pH ≈ 5.5) and lysosomes (pH ≈ 4.8), potentially leading to the production of harmful free radicals.

Lastly, the complex interplay between the AuNC surface and its stabilizing enzymes or ligands presents a significant challenge. Different factors, such as the surface charge, functional groups, and surface distance, all play crucial roles in modulating the catalytic activity of AuNCs [106]. Understanding and controlling these interactions to optimize their performance is a complex task.

Addressing these challenges will be crucial for advancing the development and application of enzyme-stabilized gold nanoclusters in various fields, from biosensing to targeted therapy and environmental remediation.

### 4.2. Recommendations for Future Research Directions

Although several issues remain to be resolved, enzyme–AuNCs hold great promise for various applications, particularly in biomedicine and biosensing, and their prospects can be significantly enhanced through specific research directions. To begin with, optimizing synthesis methods is crucial; researchers should develop more precise and scalable production techniques, such as exploring microfluidic or continuous flow systems to achieve better control over size, shape, and mono-dispersity. Additionally, investigating green synthesis methods will improve their biocompatibility and reduce their environmental impact. Improved characterization techniques are also essential; employing advanced spectroscopic and microscopic methods will help elucidate the relationship between the protein structure and the AuNC’s fluorescence properties, while techniques like thermogravimetric analysis and MALDI-TOF can provide accurate determinations of the number of gold atoms in AuNC cores. Moreover, enhancing their functionality and stability is vital for advancing AuNC applications. Research into thermostable enzyme stabilizers can lead to the development of heat-stable AuNCs with preserved functionality by investigating genetically engineered enzymes like KTQ5C and other thermostable proteins [107]. Furthermore, systematic studies on the effects of the pH and ionic strength on AuNC stability and fluorescence will enable the development of strategies to maintain their performance across a broader pH range. Furthermore, developing multiplex detection platforms capable of simultaneously detecting multiple analytes—such as hydrogen peroxide, glucose, and uric acid—will optimize ratiometric fluorescence detection methods for improved sensitivity and accuracy. Furthermore, scientific investigation of the mechanisms of enzyme–AuNC formation could help extend their applications within biosensors, guiding the artificial design to customize the biosensing mechanisms.

## 5. Conclusions

This review summarized the development of enzyme–AuNCs and their applications in biosensors, especially based on their optical and electrochemical phenomena. Also, we described the current challenges and specific recommendations for future research directions. Although significant developments have been made in the use of enzyme–AuNCs in biosensing applications, several challenges remain in terms of their wider application. However, due to excellent optical/electrochemical properties of AuNCs, such as their high fluorescence, large Stokes shift, excellent conductivity, and highly specific reactions with enzymes, enzyme–AuNCs show huge promise for highly specific and sensitive biosensing. We believe that improvements in biosensing processes that involve enzyme–AuNCs will enable the development of more efficient and sensitive biosensors with the capacity to improve well-being at the global scale.

## Figures and Tables

**Table 1:** Summary of types of enzyme–AuNCs and their applications in biosensors.

Role of Enzyme	AuNC- Stabilizing Enzyme	Target	Detection Techniques	Detection Range	LOD	Ref.
Target specificity and AuNC- stabilizing template	Horseradish peroxidase (HRP)	H_2_O_2_	Fluorescence	100 nM–100 μM	30 nM	[66]
		H_2_O_2_	Fluorescence	1–40 nM	1.0 nM	[67]
		H_2_O_2_	Fluorescence	500 pM–50 mM	0.5 nM	[68]
		Anti-PDL1 AAb l via H_2_O_2_	Colorimetry	0.2 to 1200 ng/mL	98 pg/mL	[69]
		H_2_O_2_	Electrochemical	0.01–3.27 mM	0.99 μM	[73]
		H_2_O_2_	Electrochemical	2–24 μM	443 nM	[74]
		DNA through H_2_O_2_	Electrochemical	10 aM–10 nM	8.0 aM	[75]
	Catalase (CAT)	H₂O₂	Fluorescence	10–80 μM	25 nM	
		Glucose (through GOx)	Fluorescence	5–500 μM	3 mM	[78]
		Uric acid (through UOx)	Fluorescence	10–200 μM	40 nM	
	Glucose oxidase (GOx)	H_2_O_2_	Fluorescence	0.5–10 μM	0.23 μM	[83]
		Glucose	Fluorescence	2.0–140 μM	0.7 μM	[82]
		Glucose	Fluorescence	60 μM–1.5 mM	0.03 mM	[84]
		Glucose	Electrochemical	13 μM–0.15 mM	13 μM	[87]
		Glucose	Electrochemical	5–320 nM	2.58 nM	[90]
AuNC- stabilizing template	Lysozyme (Lys)	Glutathione	Fluorescence	0.06–10 μM	20 nM	[95]
		pH	Fluorescence	7.5–9.5	-	[96]
		Folic acid	Fluorescence	0–4.76 nM	0.19 nM	[97]
	Pepsin (Peps)	Hg^2+^	Fluorescence	1–200 nM	1 nM	[99]

## Data Availability

All cited references are listed in PubMed and the Web of Science.

## Abbreviations

Abbreviation	Full Name
AuNCs	gold nanoclusters
AuNC-DENPs	AuNC-embedded dual-enzyme nanoparticles
AuNPs	gold nanoparticles
BSA	bovine serum albumin
CAT	catalase
CFUMEs	carbon fiber ultramicroelectrodes
Cys	cysteine
Enzyme–AuNCs	enzyme-stabilized gold nanoclusters
FAD	flavin adenine dinucleotide
GCE	glassy carbon electrode
GOx	glucose oxidase
GSH	glutathione
HEFBNPs	HRP–AuNC-encapsulated fluorescent bio-nanoparticles
HRP	horseradish peroxidase
H_2_O_2_	hydrogen peroxide
LOD	limit of detection
Lys	lysozyme
LysNP–AuNCs	Lysozyme nanoparticle-encapsulated AuNCs
MCN	mesoporous carbon nitride
MWCNTs	multiwalled carbon nanotubes
OVA	Ovalbumin
Peps	Pepsin
ROS	reactive oxygen species
SA–HRP–AuNCs	Streptavidin–HRP-stabilized AuNCs
TEM	transmission electron microscopy
Tyr	tyrosine
UOx	urate oxidase
